# Genomic and immune profiling of pre-invasive lung adenocarcinoma

**DOI:** 10.1038/s41467-019-13460-3

**Published:** 2019-11-29

**Authors:** Haiquan Chen, Jian Carrot-Zhang, Yue Zhao, Haichuan Hu, Samuel S. Freeman, Su Yu, Gavin Ha, Alison M. Taylor, Ashton C. Berger, Lindsay Westlake, Yuanting Zheng, Jiyang Zhang, Aruna Ramachandran, Qiang Zheng, Yunjian Pan, Difan Zheng, Shanbo Zheng, Chao Cheng, Muyu Kuang, Xiaoyan Zhou, Yang Zhang, Hang Li, Ting Ye, Yuan Ma, Zhendong Gao, Xiaoting Tao, Han Han, Jun Shang, Ying Yu, Ding Bao, Yechao Huang, Xiangnan Li, Yawei Zhang, Jiaqing Xiang, Yihua Sun, Yuan Li, Andrew D. Cherniack, Joshua D. Campbell, Leming Shi, Matthew Meyerson

**Affiliations:** 1 Department of Thoracic Surgery, Fudan University Shanghai Cancer Center, Shanghai, China; 2 Department of Oncology, Shanghai Medical College, Fudan University, Shanghai, China; 3 State Key Laboratory of Genetic Engineering, School of Life Sciences, Fudan University, Shanghai, China; 4 Institute of Thoracic Oncology, Fudan University, Shanghai, China; 5 Department of Medical Oncology, Dana-Farber Cancer Institute, Boston, MA USA; 6 Broad Institute of MIT and Harvard, Cambridge, MA USA; 7 Harvard Medical School, Boston, MA USA; 8 Computational Biology Program, Fred Hutchinson Cancer Research Center, Seattle, WA USA; 9 Human Phenome Institute, Fudan University, Shanghai, China; 10 Department of Pathology, Fudan University Shanghai Cancer Center, Shanghai, China; 11 Division of Computational Biomedicine, Department of Medicine, Boston University, Boston, MA USA

**Keywords:** Cancer, Genetics

## Abstract

Adenocarcinoma in situ and minimally invasive adenocarcinoma are the pre-invasive forms of lung adenocarcinoma. The genomic and immune profiles of these lesions are poorly understood. Here we report exome and transcriptome sequencing of 98 lung adenocarcinoma precursor lesions and 99 invasive adenocarcinomas. We have identified *EGFR*, *RBM10*, *BRAF*, *ERBB2*, *TP53*, *KRAS*, *MAP2K1* and *MET* as significantly mutated genes in the pre/minimally invasive group. Classes of genome alterations that increase in frequency during the progression to malignancy are revealed. These include mutations in *TP53*, arm-level copy number alterations, and HLA loss of heterozygosity. Immune infiltration is correlated with copy number alterations of chromosome arm 6p, suggesting a link between arm-level events and the tumor immune environment.

## Abstract (web summary)

The genomic and immune landscape of pre-invasive lung adenocarcinoma is poorly understood. Here, the authors perform exome and transcriptome sequencing on precursor legions and invasive lung adenocarcinomas, identifying recurrently mutated genes in pre/minimally invasive cases, and arm level alteration events linked to immune infiltration.

## Introduction

Lung adenocarcinoma (LUAD) is the most common histological subtype of lung cancer, with an average 5-year survival rate of 15%^1,2^. In contrast, the pre-invasive stages of LUAD, such as adenocarcinoma in situ (AIS) and minimally invasive adenocarcinoma (MIA), are associated with a nearly 100% survival rate, after surgical resection^3–5^. AIS is defined as a ≤3 cm adenocarcinoma lacking invasion, while MIA is a ≤3 cm adenocarcinoma with ≤5 mm invasion^6^. Although some focused studies have identified mutations in lung cancer drivers in AIS and MIA^7–10^, there remains a lack of deep insight into the molecular events driving progression of these lesions to invasive LUAD. To address this gap in our knowledge of AIS/MIA pathogenesis, we undertook a systematic investigation of the genomic and immune profiles of pre/minimally invasive lung lesions. Known driver mutations are present in the lung precursors. T cell and B cell responses to the AIS/MIA samples are observed. By comparing the genomic landscapes of the pre-invasive and invasive samples, we suggest the potential molecular events underlying the invasiveness of LUAD.

## Results

### The landscape of somatic alterations in AIS and MIA

**Fig. 1:**
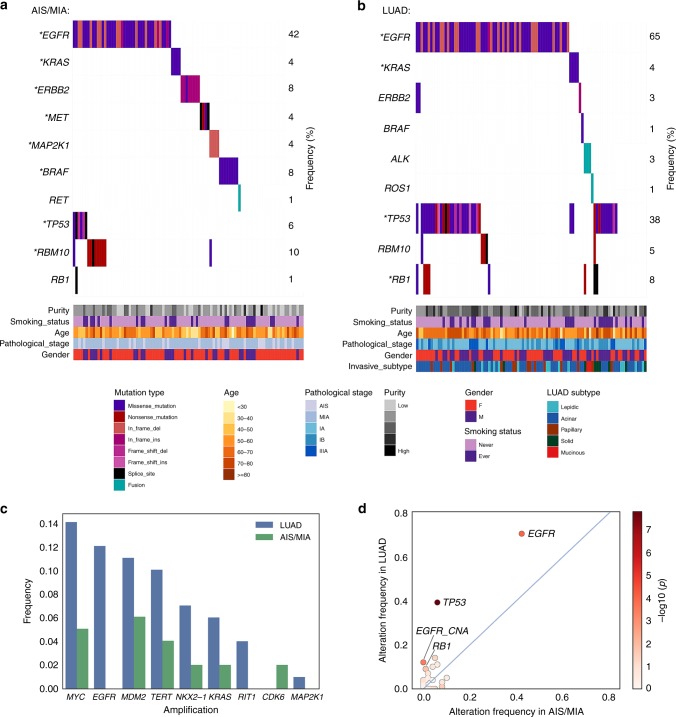
Somatic alterations in pre-invasive and invasive lung adenocarcinomas. **a** Co-mutation plots for AIS/MIA and **b** LUAD. Stars indicate significantly mutated genes in each group. **c** Lung cancer genes with focal amplification in AIS/MIA and LUAD. **d** Somatic alterations with higher frequencies in LUAD, compared to AIS and MIA. Color bar represents log_10_-transformed *p* value calculated from two-sided Fisher’s exact test. Source data are provided as a source data file.

We performed whole-exome sequencing (WES) and RNA-sequencing (RNA-seq) on tumor and matched adjacent normal tissue of 24 AIS, 74 MIA, and 99 invasive LUAD samples (Supplementary Table 1), obtained from patients who underwent surgery at Fudan University Shanghai Cancer Center (FUSCC). We identified eight significantly mutated genes in AIS and MIA specimens, including *EGFR*, *RBM10*, *BRAF*, *ERBB2*, *TP53*, *KRAS*, *MAP2K1*, and *MET*, all previously reported as recurrently mutated in LUAD from The Cancer Genome Atlas (TCGA) cohort^11,12^. *EGFR*, *TP53*, *RB1*, and *KRAS* were significantly mutated in the tested LUAD cases (Fig. 1a, b). Amplified regions that included *MDM2*, *MYC*, *TERT*, *KRAS*, *NKX2-1*, and *CDK6* were observed in the AIS or MIA samples (Fig. 1c). Novel amplifications of *RIT1* were identified in the FUSCC LUAD cohort (Supplementary Fig. 1). RNA-seq analysis revealed a *RET* fusion in an MIA sample (Fig. 1a), and *ALK* and *ROS1* fusions in LUAD (Fig. 1b). When testing significantly mutated genes, *TP53* mutations were the most enriched alteration in the invasive stage (38%) compared to pre/minimally invasive stages (6%), followed by *EGFR* and *RB1* mutations (Fig. 1d). When testing all mutated genes in the pre/minimally invasive lung lesions, only *TP53* mutations significantly increased in frequency through malignancy, after false discovery rate correction.

### The relatively simpler genomes in AIS and MIA than LUAD

Tumor mutation burden (TMB) was significantly lower in AIS and MIA, compared to stage I LUAD (Supplementary Fig. 2a). Mutational signature analysis identified aging, smoking, APOBEC, and DNA mismatch repair signatures in our cohort. The APOBEC signature was higher in MIA compared to LUAD, although the smoking signature activity did not differ among the three groups (Supplementary Fig. 2b, c). Arm-level copy-number alteration (CNA) was less common in the pre/minimally invasive stages, with a median of 5, 11, and 26 events in AIS, MIA, and LUAD, respectively (Supplementary Fig. 3a). Similarly, focal CNA increased from MIA to LUAD (Supplementary Fig. 3b). TMB, arm-level CNA and focal CNA were all correlated with advancing malignant potential, controlling for specimen purity (linear regression, *p* < 0.001, Methods, Supplementary Fig. 4a, b).

### Molecular mechanism underlying the invasive progression

**Fig. 2:**
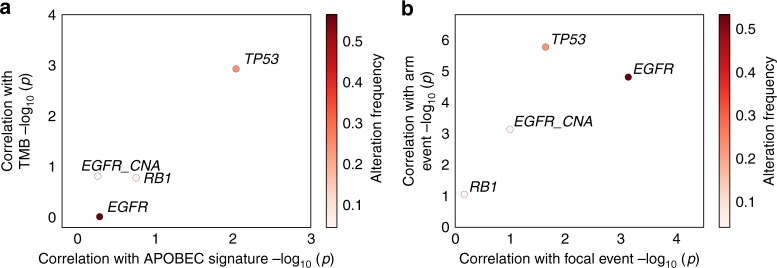
Correlation of somatic alterations with genomic features. *TP53*, *EGFR*, *RB1* mutations and *EGFR* amplification in correlation with **a** TMB and APOBEC signature, and **b** arm and focal CNA. Student’s *t* test was used to calculate the log_10_-transformed *p* value. Samples in all stages were included to calculate the alteration frequency. Source data are provided as a source data file.

Next, we tested the association of genes with increased alteration frequency from AIS/MIA to LUAD and genomic features that distinguish LUAD from AIS/MIA (increased TMB, APOBEC signature, and focal and arm-level CNAs). Notably, *TP53* mutations were strongly correlated with arm-level and TMB, but marginally correlated with focal CNA events (Fig. 2a, b). These data suggest that, in contrast to oncogenic mutations, which occurred frequently in pre/minimally invasive lung tumors, *TP53* mutations were highly involved in the invasiveness during tumor development.

### Immune characterization of AIS and MIA

In the analysis of T cell receptor (TCR) repertoire and B cell receptor (BCR) repertoire, we observed a tendency that the highest-frequency T cell clones or B cell clones in the tumors were represented as lower frequency clones in the matched normal tissues (Supplementary Fig. 5a, b). However, neither T cell nor B cell clonality was increased from normal samples to AIS/MIA or LUAD (Supplementary Fig. 6a, b).

**Fig. 3:**
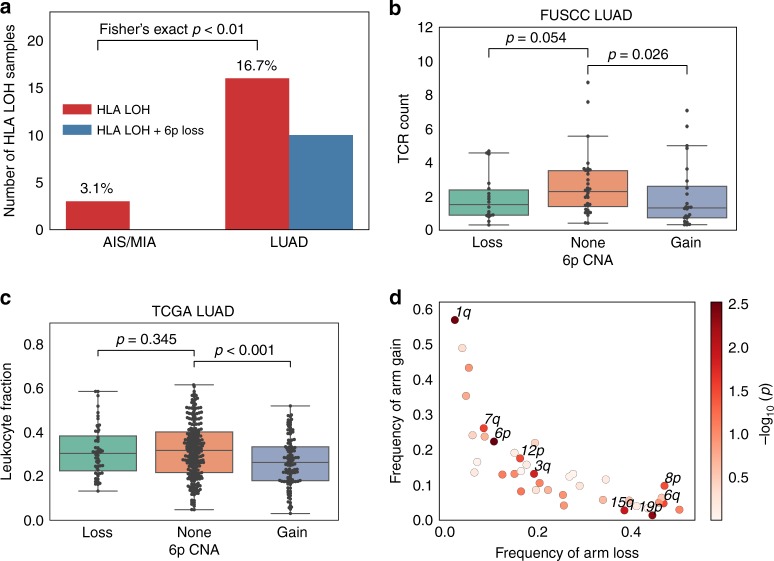
Tumor immune environment in association with arm-level CNA. **a** Frequency of loss of HLA heterozygosity and the co-occurrence of HLA LOH with 6p loss. Significantly more HLA LOH events are found in the LUAD group compared to the AIS/MIA group. **b** Comparison of inferred T cell infiltration in FUSCC LUAD samples and **c** leukocyte infiltration^15^ in TCGA LUAD samples with 6p CNA loss, gain, or no change. *P* values are calculated from Mann–Whitney *U* test. Significantly decreased level of T cell or leukocyte infiltrations are found in 6p gain samples compared to 6p neutral samples. In the box plots, the upper and lower hinges represent the first and third quartile, the whiskers span the first and third quartile, and center lines represent the median. **d** Correlation of arm-level CNA with leukocyte infiltration for the TCGA LUAD samples. *P* values are calculated from multivariate linear regression, while each arm is assigned 1 if gained, −1 if lost and 0 if unchanged, and adding the aneuploidy score^18^ and TMB as covariates. Source data are provided as a source data file.

Loss of human leukocyte antigen (HLA) alleles has been identified as a potential immune escape mechanism in lung cancers^13,14^ and can be observed as a subclonal event in LUADs^14^. In our study, we noted HLA loss of heterozygosity (LOH) in 3.1% of AIS/MIA and 16.7% of LUAD specimens (Fig. 3a). The significantly increased frequency of HLA LOH in the invasive group compared to the pre-invasive group (Fisher’s exact test, *p* < 0.01) suggested the potential role of loss of HLA alleles during tumor development. The frequency of germline HLA homozygosity, however, was similar in all three stages (Supplementary Fig. 7a). Approximately 60% of the HLA LOH events in LUAD were related to loss of chromosome 6p. Interestingly, we found that 6p gain was significantly anti-correlated with T cell abundance (Mann–Whitney *U* test, *p* = 0.038, Fig. 3b), and this trend was also observed when analyzing B cell infiltration in correlation with 6p CNA (Supplementary Fig. 7b–d). We subsequently tested the correlation of immune infiltration with large-scale chromosome alterations, using samples from the TCGA LUAD cohort. We observed the most significant correlation of leukocyte fraction^15^ with chromosome 6p CNA (*p* = 0.0030, coef. = −0.74, 95% CI: −1.23 to −0.25), followed by 1q (*p* = 0.0033, coef. = −0.60, 95% CI: −1 to −0.2) and 19p CNA (*p* = 0.0047, coef. = 0.53, 95% CI: 0.16 to 0.9), after controlling for TMB and the degree of overall aneuploidy (see Methods, Fig. 3c, d). 6p and 1q CNA showed significantly increased frequency from AIS/MIA to LUAD in the FUSCC cohort (Fisher’s exact test, *p* < 0.001, Supplementary Fig. 7e).

## Discussion

We have interrogated the genomic and immune features of pre/minimally invasive lung cancers. Seventy-one percent of AIS and MIA patients carried at least one mutation in previously identified cancer genes in the RTK/RAS/RAF pathway, similar to the oncogenic driver events found in LUAD. In addition, we showed an overall high frequency of *EGFR* mutations (65% in LUAD), which may reflect the enrichment of never smoking patients with East Asian origin in our cohort. APOBEC-related mutations are contributors to lung cancer heterogeneity^16^, and might be involved in the progression from AIS/MIA to LUAD^10^. We found that genomic aberrations including TMB, APOBEC signature, and arm and focal CNA were increased from the pre-invasive to invasive stage. Mutations in *TP53* and HLA LOH also increased in frequency in the aggressive stage .

Our work reveals *TP53* as a key mediator in the invasiveness of lung cancer. Previous studies in Barrett’s esophagus suggested that *TP53* occurred early in esophageal adenocarcinoma precursors followed by oncogenic amplifications^17^. *TP53* was also frequently mutated in lung carcinoma in situ, which is the precursor form of squamous cell carcinoma^18^. We have shown the high frequency of oncogenic driver mutations, but low frequency of *TP53* mutations in the LUAD precursors. Previous studies have suggested the functional association of *TP53* mutations with invasive potential in cancers^19^. Our findings also demonstrate a strong association of *TP53* mutations with aneuploidy, in line with recent work from TGCA^20^. Given previous reports of aneuploidy in association with decreased immune infiltration^20,21^, our data raise the possibility that copy-number changes in specific chromosomes may influence the tumor microenvironment. Our work provides new insights into the biology of lung pre-malignancy, with implications for disease monitoring and prognosis, and future therapeutic intervention.

## Methods

### Patient cohort and pathological review

One hundred and ninety-seven patients who underwent surgery between September 2011 and May 2016 at the Department of Thoracic Surgery, Fudan University Shanghai Cancer Center were enrolled in this study. No patient received neoadjuvant therapy. Preoperative tests, including contrast-enhanced chest computed tomography (CT) scanning, were performed to determine the clinical stage of the disease. Fiber optic bronchoscopy was routinely performed. When necessary, CT-guided hook-wire localization was performed before surgery, to define the resection area. Tumor specimens were initially sent for intraoperative frozen section diagnosis after they were removed. The specimen was sliced at the largest diameter of the tumor for sampling. Usually two sections of each specimen were made for intraoperative diagnosis. After surgery, the tumor specimens were sent to be reviewed by two pathologists independently to confirm the clinical stage and determine the histological classification. Stage IIIA patients in this study cohort were those with initial clinical stage I diagnosis, but mediastinal lymph node metastasis was found by postsurgical pathological review. Usually 3–5 sections of each specimen were used to determine the final pathological diagnosis. Tumors were classified into AIS, MIA, and invasive adenocarcinoma, according to the LUAD classification of the International Association for the Study of Lung Cancer, American Thoracic Society, and European Respiratory Society^1^. For invasive adenocarcinomas, the occupancy of each one of these several patterns, namely, lepidic, acinar, papillary, micropapillary, solid, and invasive mucinous adenocarcinoma, was recorded in a 5% increment, and the subtype with the highest percentage was considered as the predominant subtype. This study was approved by the Committee for Ethical Review of Research (Fudan University Shanghai Cancer Center Institutional Review Board No. 090977-1). Informed consents of all patients for donating their samples to the tissue bank of Fudan University Shanghai Cancer Center were obtained from patients themselves or their relatives. Source data are provided as a source data file.

### Whole-exome sequencing

Genomic DNA from tumors and paired adjacent normal tissues was extracted and prepared using the QIAamp DNA Mini Kit (Qiagen) following the manufacturer’s instructions. Exon libraries were constructed using the SureSelect XT Target Enrichment System. A total amount of 1–3 µg genomic DNA for each sample was fragmented into an average size of ~200 bp. DNA was captured using SureSelect XT reagents and protocols to generate indexed, target-enriched library amplicons. Constructed libraries were then sequenced on the Illumina HiSeq X Ten platform and 150 bp paired-end reads were generated.

### RNA-sequencing

Total RNA from tumors and paired adjacent normal tissues was extracted and prepared using NucleoZOL (Macherey-Nagel) and NucleoSpin RNA Set for NucleoZOL (Macherey-Nagel) following the manufacturer’s instructions. A total amount of 3 µg RNA per sample was used as initial material for RNA sample preparations. Ribosomal RNA was removed using Epicenter Ribo-Zero Gold Kits (Epicenter, USA). Subsequently, the sequencing libraries were generated using the NEBNext Ultra Directional RNA Library Prep Kit for Illumina (NEB, Ipswich, USA) according to manufacturer’s instructions. Libraries were then sequenced on the Illumina HiSeq X Ten platform and 150 bp paired-end reads were generated.

### Alignment and mutation calling

Sequencing reads from the exome capture libraries were aligned to the reference human genome (hg19) using BWA-MEM^22^. The Picard tools (https://broadinstitute.github.io/picard/) was used for marking PCR duplicates. The Genome Analysis Toolkit^23^ was used to perform base quality recalibration and local indel re-alignments. SNVs were called using MuTect and MuTect2^24^. Indels were called using MuTect2 and Strelka v2.0.13^25^. Variants were filtered if called by only one tool. Oncotator v1.9.1^26^ was used for annotating somatic mutations. Significantly mutated genes were identified using MutSig2CV^27^. TMB was calculated as the total number of nonsynonyous SNVs and indels per sample divided by 30, given coverage of ~30 MB. Linear regression was used to test the correlation of TMB with disease stages, while coding AIS, MIA, and LUAD as 0, 1, and 2, respectively, and adding purity as a covariate.

### Mutational signature and copy-number changes

Mutational signature was called using SignatureAnalyzer^28^ with SNVs classified by 96 tri-nucleotide mutation. Read coverage was calculated at 50 kb bins across the genome and was corrected for GC content and mappability biases using ichorCNA v0.1.0^29^. The copy-number analysis was performed using TitanCNA v1.17.1^30^. GISTIC 2.0.22^31^ was used to identify amplification peaks and to separate arm and focal level CNA using ichorCNA generated segments. Arm-level event was defined by log_2_-transformed copy-number ratio >0.1 or <−0.1. Focal level events were defined by log_2_-transformed copy-number ratios of >1 or <−1. For *EGFR* and *KRAS* in the AIS/MIA samples, we lowered the amplification threshold to 0.8, and did not detect additional events. Purity and ploidy were calculated by the ABSOLUTE algorithm^32^. Linear regression was used to test the correlation of focal and arm-level CNA with disease stages, while coding AIS, MIA, and LUAD coded as 0, 1, and 2, respectively, and adding purity as a covariate.

### Analysis of expression and fusion

RNA-seq reads were aligned to the reference human genome (hg19) with STAR v2.5.3^33^. Expression values were normalized to the transcripts per million (TPM) estimates using RSEM v1.3.0^34^. The log_2_-transformed TPM values were used to measure gene expression. Fusion events were called using STAR-fusion^35^. We focused on known lung cancer fusions (*ALK*, *ROS1*, *NTRK2*, *RET*, and *MET*) with read count supporting the fusion event >10, and visually inspected the BAM files to ensure accuracy.

### TCR, BCR, and HLA analysis

TCR or BCR sequences were analyzed using MiXCR 2.1.11^36^ based on the RNA-seq data. The reads per million (RPM) value was used to normalize the total TCR or BCR count to the total reads aligned in sample. Infiltration was inferred by the RPM of TCR or BCR count. T cell or B cell diversity is inferred by the Shannon entropy score. Samples that have at least 10 clones with clone count >5 were used in the entropy test. For each sample, we calculated the entropy score based on the top 10 clones. Samples with purity <0.2 and >0.8 were excluded. Samples with possible contamination (top clones found in more than one samples) were excluded. HLA types were called with POLYSOLVER^37^. Loss of HLA heterozygosity was called by LOHHLA^14^. An event of the copy number calculated with binned B-allele frequency <0.5 and the *p* value (Pval_unique) of allelic imbalance <0.1 was considered as HLA LOH for AIS or MIA, and 0.05 for LUAD. For the analyses with TCGA samples, we obtained the fraction of leukocytes, TMB, aneuploidy score, and arm-level CNA from Taylor et al.^20^. Linear regression was used to test the correlation of arm CNA with the leukocyte fraction, while coding loss, gain, and none as −1, 1, and 0, respectively, and adding TMB and aneuploidy score as covariates.

### Reporting summary

Further information on research design is available in the Nature Research Reporting Summary linked to this article.

## Supplementary information

Supplementary Information

Reporting Summary

## Data Availability

Raw data from WES and RNA-seq of AIS/MIA and LUAD have been deposited at European Genome-phenome Archive (EGA) under the accession code EGAS00001004006. Source data underlying all figures are provided as a Source Data file.

## Source Data

Source Data

## Code availability

All custom code used in the analyses is available at https://github.com/jcarrotzhang/Code-for-preinvasive.

## References

[1] Siegel RL (2018). Cancer statistics, 2018. CA Cancer J. Clin..

[2] Chen W (2016). Cancer statistics in China, 2015. CA Cancer J. Clin..

[3] Yim J (2007). Histologic features are important prognostic indicators in early stage lung adenocarcinomas. Mod. Pathol..

[4] Borczuk AC (2009). Invasive size is an independent predictor of survival in pulmonary adenocarcinoma. Am. J. Surg. Pathol..

[5] Maeshima AM (2010). Histological scoring for small lung adenocarcinomas 2 cm or less in diameter: a reliable prognostic indicator. J. Thorac. Oncol..

[6] Travis WD (2011). International association for the study of lung cancer/American thoracic society/European respiratory society international multidisciplinary classification of lung adenocarcinoma. J. Thorac. Oncol..

[7] Murphy SJ (2014). Genomic rearrangements define lineage relationships between adjacent lepidic and invasive components in lung adenocarcinoma. Cancer Res..

[8] Izumchenko E (2015). Targeted sequencing reveals clonal genetic changes in the progression of early lung neoplasms and paired circulating DNA. Nat. Commun..

[9] Kobayashi Y (2015). Genetic features of pulmonary adenocarcinoma presenting with ground-glass nodules: the differences between nodules with and without growth. Ann. Oncol..

[10] Vinayanuwattikun C (2016). Elucidating genomic characteristics of lung cancer progression from in situ to invasive adenocarcinoma. Sci. Rep..

[11] The Cancer Genome Atlas Research Network. (2014). Comprehensive molecular profiling of lung adenocarcinoma. Nature.

[12] Campbell JD (2016). Distinct patterns of somatic genome alterations in lung adenocarcinomas and squamous cell carcinomas. Nat. Genet..

[13] The Cancer Genome Atlas Research Network. (2012). Comprehensive genomic characterization of squamous cell lung cancers. Nature.

[14] McGranahan N (2017). Allele-specific HLA loss and immune escape in lung cancer evolution. Cell.

[15] Thorsson V (2018). The immune landscape of cancer. Immunity.

[16] de Bruin EC (2014). Spatial and temporal diversity in genomic instability processes defines lung cancer evolution. Science.

[17] Stachler MD (2015). Paired exome analysis of Barrett’s esophagus and adenocarcinoma. Nat. Genet..

[18] Teixeira VH (2019). Deciphering the genomic, epigenomic, and transcriptomic landscapes of pre-invasive lung cancer lesions. Nat. Med..

[19] Goh AM (2011). The role of mutant p53 in human cancer. J. Pathol..

[20] Taylor AM (2018). Genomic and functional approaches to understanding cancer aneuploidy. Cancer Cell.

[21] Davoli T (2017). Tumor aneuploidy correlates with markers of immune evasion and with reduced response to immunotherapy. Science.

[22] Li, H. Aligning sequence reads, clone sequences and assembly contigs with BWA-MEM. Preprint at https://arxiv.org/abs/1303.3997 (2013).

[23] DePristo MA (2011). A framework for variation discovery and genotyping using next-generation DNA sequencing data. Nat. Genet..

[24] Cibulskis K (2013). Sensitive detection of somatic point mutations in impure and heterogeneous cancer samples. Nat. Biotechnol..

[25] Saunders CT (2012). Strelka: accurate somatic small-variant calling from sequenced tumor-normal sample pairs. Bioinformatics.

[26] Ramos AH (2015). Oncotator: cancer variant annotation tool. Hum. Mutat..

[27] Lawrence MS (2014). Discovery and saturation analysis of cancer genes across 21 tumour types. Nature.

[28] Kim J (2016). *Somatic ERCC2 mutations are associated with a distinct genomic signature in urothelial tumors*. Nat. Genet..

[29] Adalsteinsson VA (2017). Scalable whole-exome sequencing of cell-free DNA reveals high concordance with metastatic tuomrs. Nat. Commun..

[30] Ha G (2014). TITAN: inference of copy number architectures in clonal cell populations from tumor whole-genome sequence data. Genome Res..

[31] Mermel CH (2011). GISTIC2.0 facilitates sensitive and confident localization of the targets of focal somatic copy-number alteration in human cancers. Genome Biol..

[32] Carter SL (2012). Absolute quantification of somatic DNA alterations in human cancer. Nat. Biotechnol..

[33] Dobin A (2013). STAR: ultrafast universal RNA-seq aligner. Bioinformatics.

[34] Li B, Dewey CN (2011). RSEM: accurate transcript quantification from RNA-Seq data with or without a reference genome. BMC Bioinformatics.

[35] Brian, J. H. et al. STAR-Fusion: fast and accurate fusion transcript detection from RNA-seq. Preprint at https://www.biorxiv.org/content/early/2017/03/24/120295 (2017).

[36] Bolotin DA (2015). MiXCR: software for comprehensive adaptive immunity profiling. Nat. Methods.

[37] Shukla SA (2015). Comprehensive analysis of cancer-associated somatic mutations in class I HLA genes. Nat. Biotechnol..

