# Supplementary material for: Genomic and immune profiling of pre-invasive lung adenocarcinoma
Source: Nat Commun. 2019 Nov 29;10:5472. doi: 10.1038/s41467-019-13460-3 (PMC6884501; doi:10.1038/s41467-019-13460-3)
Supplement: Supplementary file 1 — Supplementary Information [file 41467_2019_13460_MOESM1_ESM.pdf]

Supplemental Information

Genomic and immune profiling of pre-invasive lung adenocarcinoma

Chen, Carrot-Zhang, Zhao et al.

Supplementary Figures

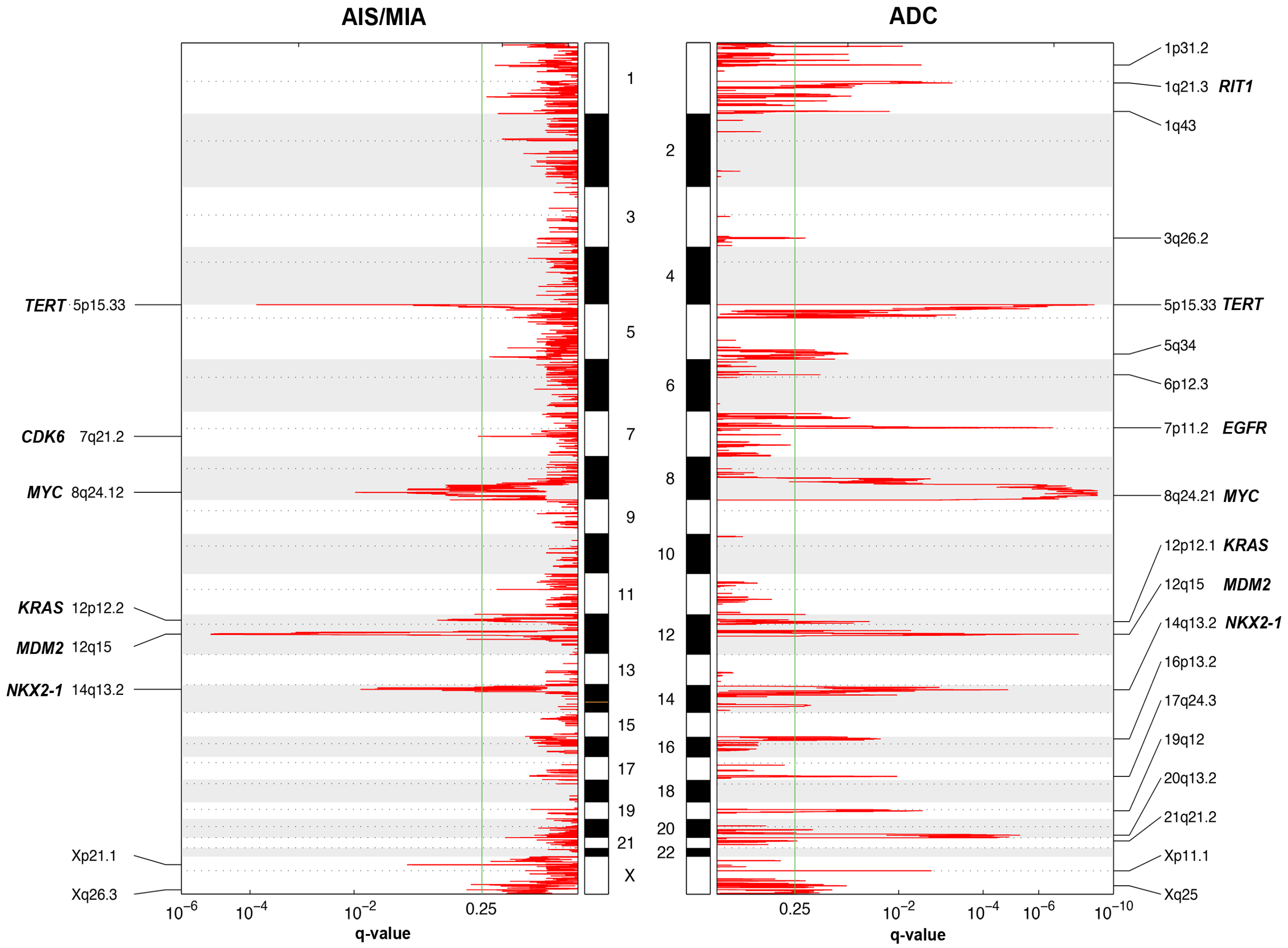

Supplementary Figure 1: **CNA profile of AIS/MIA and LUAD.** We applied GISTIC2.0 to identify significant amplification peaks based on ichorCNA generated copy number profiles in the AIS/MIA group and LUAD group, respectively. Samples with greater than 2500 segments (11 AIS/MIA and 3 LUAD samples) are excluded in this discovery phase. Source data are provided as a Source Data file.

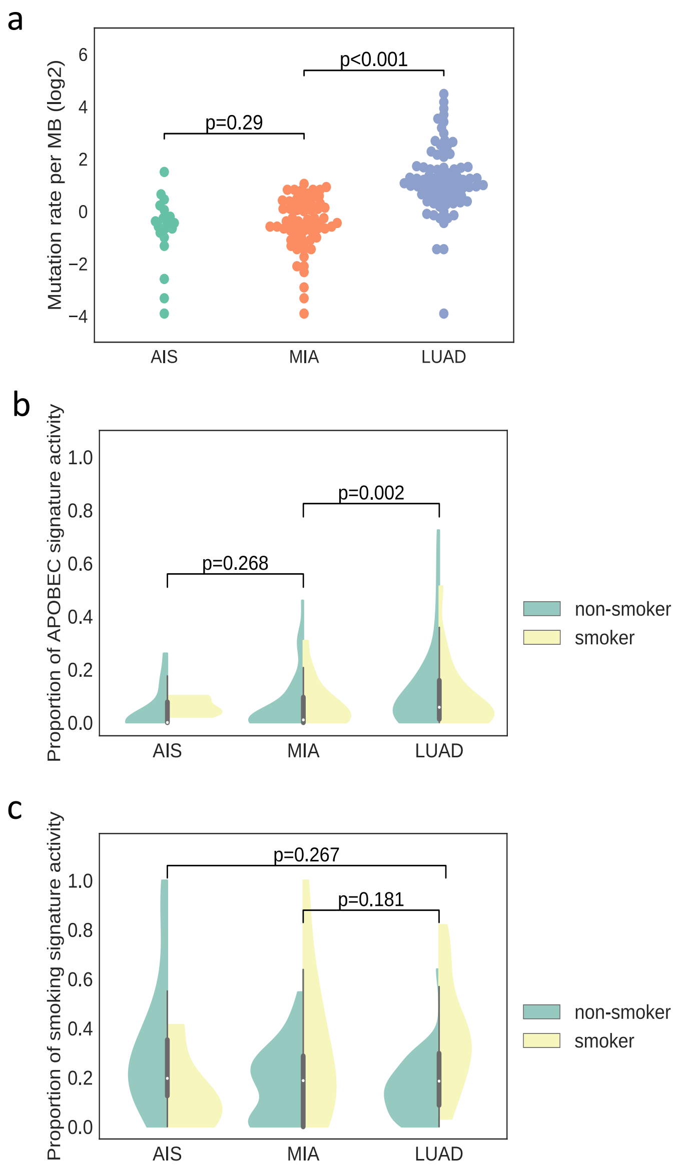

Supplementary Figure 2: **TMB, smoking and APOBEC signature in AIS/MIA and LUAD.** (a) Comparison of TMB in AIS, MIA patients and LUAD patients with significantly higher TMB in the LUAD group. AIS, MIA and stage I LUAD patients are included in this analysis. 13 samples without estimated purity are excluded. (b) Comparison of the proportion of mutations with APOBEC signature and (c) with smoking signature, with significantly higher level of APOBEC signature in the LUAD group. The white dots represent the mean proportion of each signature activity, with 95% confidence interval represented as error bars. Self-reported never smokers and smokers are colored in green and yellow, respectively. AIS, MIA or stage I LUAD patients are included in this analysis. Signature calls are not available for 6 samples. P values are calculated from Mann-Whitney U test. Source data are provided as a Source Data file.

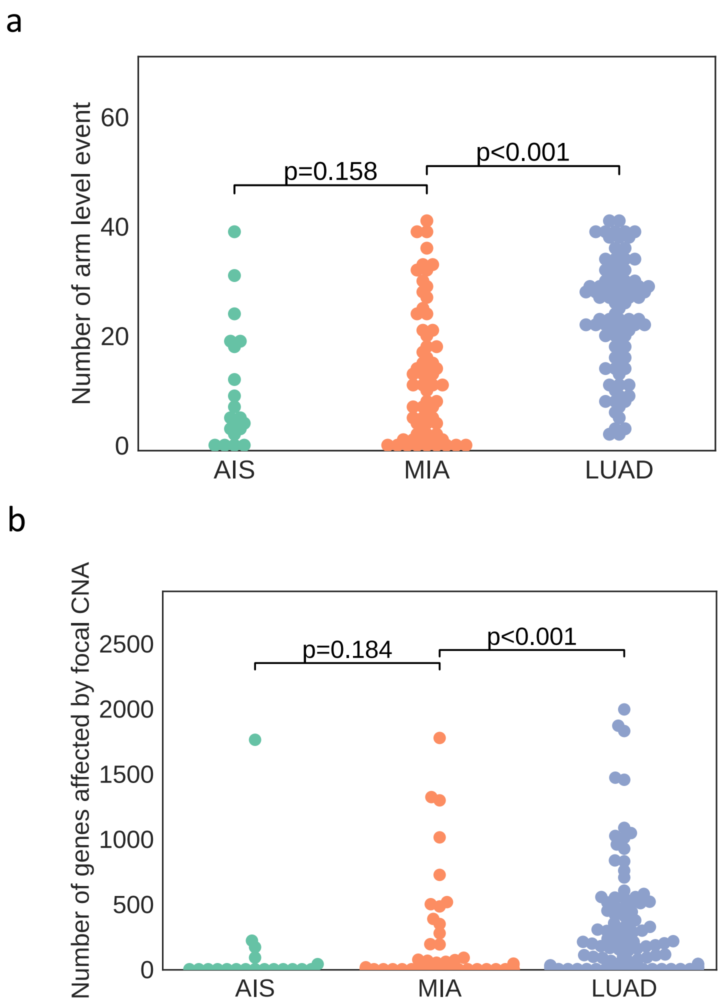

Supplementary Figure 3: **Arm and focal CNA in AIS/MIA and LUAD.** (a) Comparison of arm-level CNA and (b) number of genes affected by focal CNA in AIS, MIA and LUAD patients, with significantly higher number of arm-level and focal level CNAs in the LUAD group. Two samples with greater than 3500 segments and 13 samples without estimated purity are excluded in the arm-level CNA analysis. Additional 8 samples with more than 2500 genes affected by focal CNA are excluded in the focal CNA analysis. P values are calculated from Mann-Whitney U test. Source data are provided as a Source Data file.

a

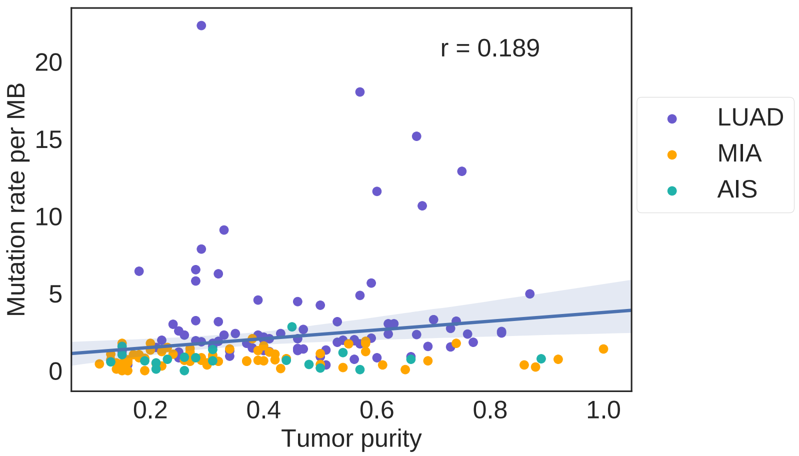

b

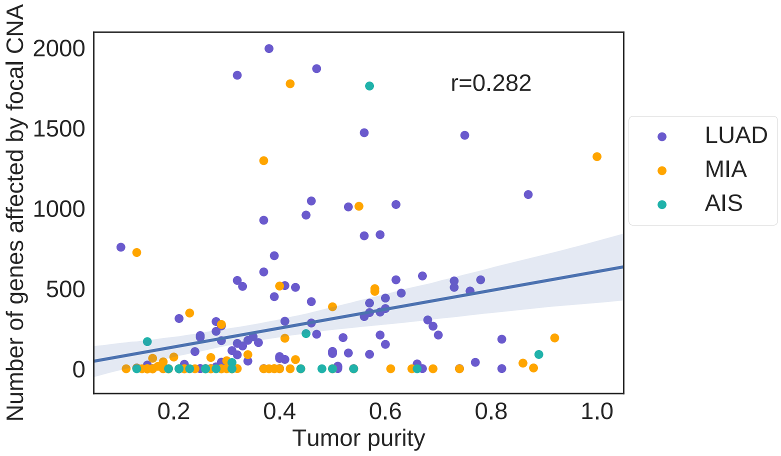

Supplementary Figure 4: Scatter plot of tumor purity (a) with TMB (n=168) and (b) with number of genes affected by focal CNA (n=174). The r values are correlation coefficient from linear regression. Source data are provided as a Source Data file.

a

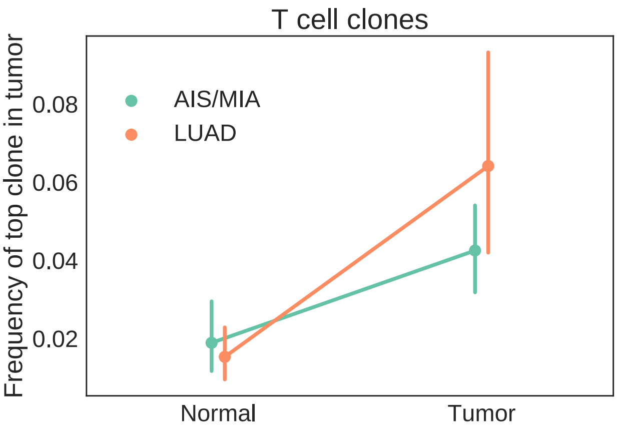

b

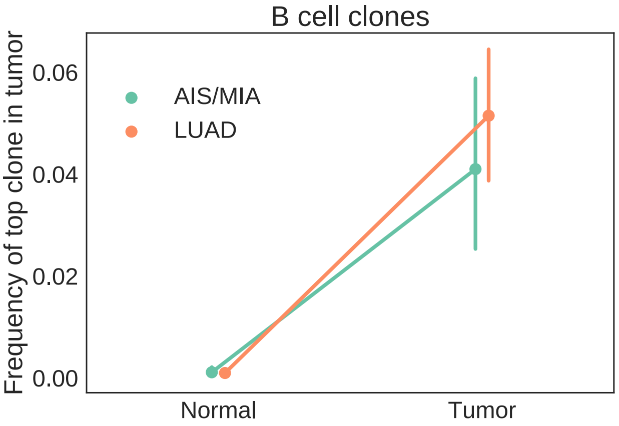

Supplementary Figure 5: **T cell and B cell top clone frequency in AIS/MIA and LUAD.** (a) Significantly increased frequencies of the top T cell clones in AIS/MIA (n=14, paired t test p=0.001) or LUAD (n=23, paired t test p=0.007) compared to their frequencies in the matched normal tissue. (b) Significantly increased frequencies of the top B cell clones in AIS/MIA (n=18, paired t test p=0.04) or LUAD (n=27, paired t test p<0.001) compared to their frequencies in the matched normal tissue. The mean frequency is represented by dots, and the 95% confidence interval is represented as error bars. Source data are provided as a Source Data file.

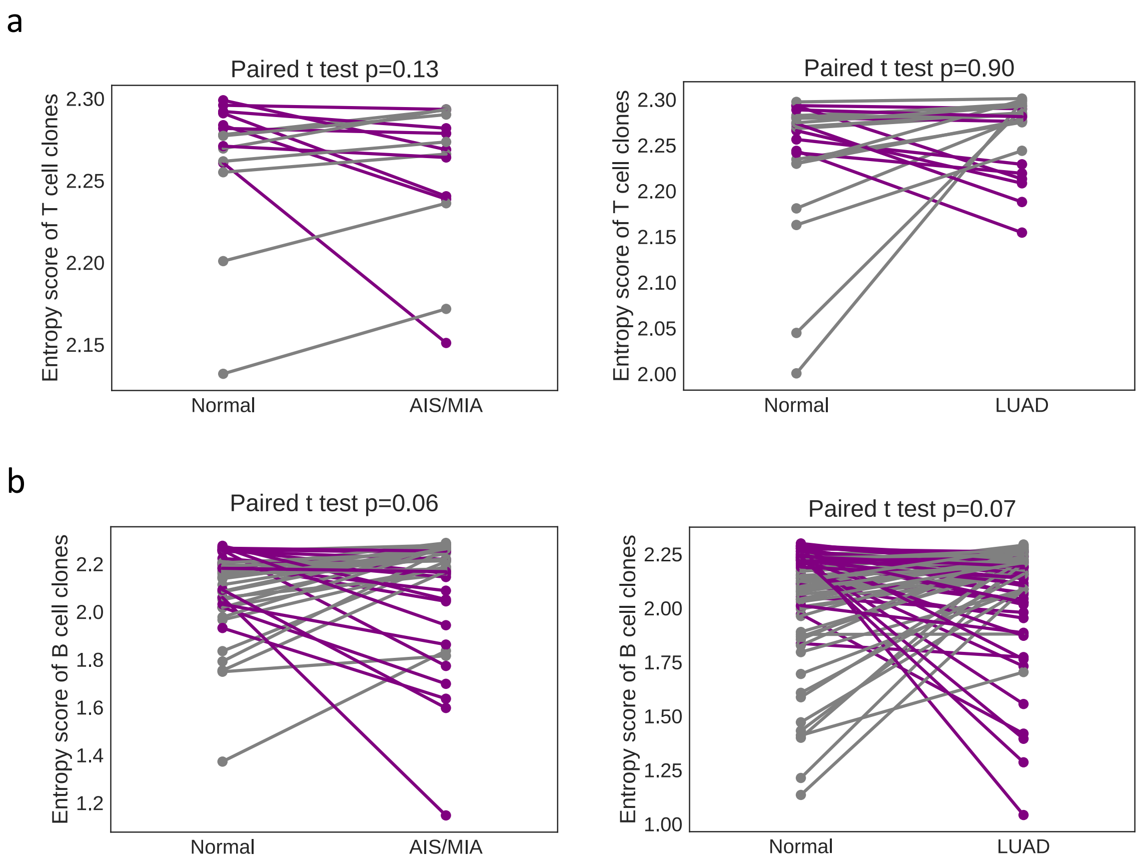

Supplementary Figure 6: **T cell and B cell clonality in AIS/MIA and LUAD.** (a) Change of entropy score from normal to AIS/MIA (left) and from normal to LUAD (right) in the T cell diversity analysis. (b) Change of entropy score from normal to AIS/MIA (left) and from normal to LUAD (right) in the B cell diversity analysis. Tumors with increased clonality inferred by decreased entropy score are highlighted in purple. No significant increase of T cell or B cell clonality from normal tissue to tumor is observed. Source data are provided as a Source Data file.

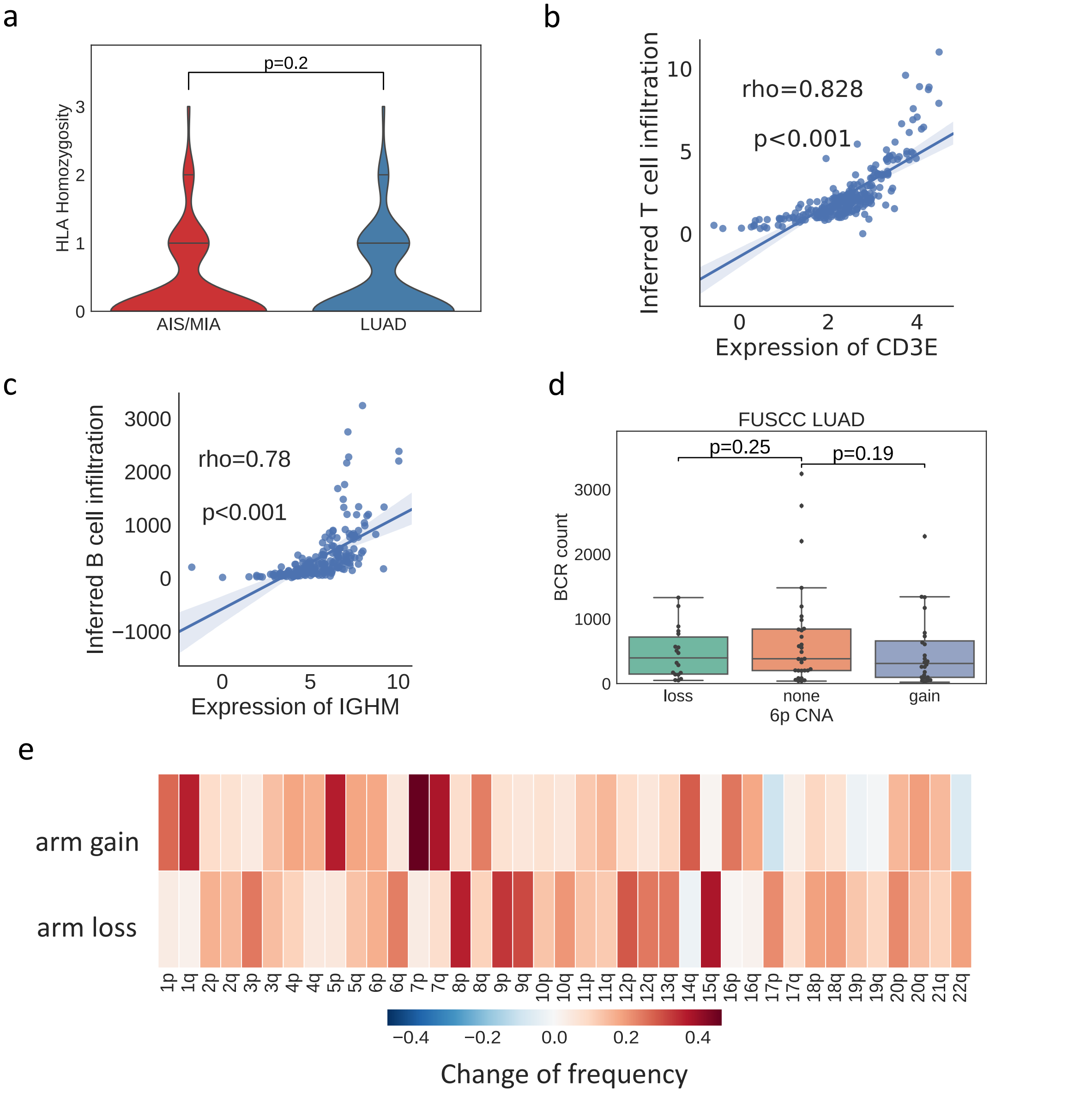

Supplementary Figure 7: **Interaction of genomic and immune features in AIS/MIA and LUAD.** (a) Comparison of HLA I (HLA-A/B/C) homozygosity between AIS/MIA and LUAD. P values are calculated from Mann-Whitney U test. T cell and B cell infiltrations are accurately inferred, as suggested in (b) high Spearman's correlation of inferred T cell infiltration with the expression of the T cell marker *CD3E* and in (c) high Spearman's correlation of inferred B cell infiltration with the expression of the B cell marker *IGHM*. (d) Comparison of inferred B cell infiltration with 6p loss, gain or no change in FUSCC LUAD samples. P values are calculated from Mann-Whitney U test. (e) Frequency change of arm-level copy number gain or loss from AIS/MIA to LUAD in FUSCC samples. Source data are provided as a Source Data file.

Supplementary Table 1: clinical characteristics of the patient cohort. Source data are provided as a Source Data file.

	AIS (24)	MIA (74)	LUAD (99)	Total (197)
Gender				
Female	17	51	52	120
Male	7	23	47	77
Pathological stage				
IA			56	
IB			27	
IIIA			16	
Smoking status				
Former/current	5	19	30	54
Never	19	55	69	143
Age (Average)	55.9	55.3	61.7	58.6
