# Supplementary material for: Genomic and immune profiling of pre-invasive lung adenocarcinoma
Source: Nat Commun. 2019 Nov 29;10:5472. doi: 10.1038/s41467-019-13460-3 (PMC6884501; doi:10.1038/s41467-019-13460-3)
Supplement: Supplementary file 2 — Reporting Summary [file 41467_2019_13460_MOESM2_ESM.pdf]

nature research

Corresponding author(s): Haiquan Chen
Last updated by author(s): 2019-9-29

# Reporting Summary

Nature Research wishes to improve the reproducibility of the work that we publish. This form provides structure for consistency and transparency in reporting. For further information on Nature Research policies, see Authors & Referees and the Editorial Policy Checklist.

## Statistics

For all statistical analyses, confirm that the following items are present in the figure legend, table legend, main text, or Methods section.

| n/a | Confirmed | |
|---|---|---|
| ☐ | ☒ | The exact sample size (*n*) for each experimental group/condition, given as a discrete number and unit of measurement |
| ☐ | ☒ | A statement on whether measurements were taken from distinct samples or whether the same sample was measured repeatedly |
| ☐ | ☒ | The statistical test(s) used AND whether they are one- or two-sided<br>*Only common tests should be described solely by name; describe more complex techniques in the Methods section.* |
| ☐ | ☒ | A description of all covariates tested |
| ☐ | ☒ | A description of any assumptions or corrections, such as tests of normality and adjustment for multiple comparisons |
| ☐ | ☒ | A full description of the statistical parameters including central tendency (e.g. means) or other basic estimates (e.g. regression coefficient) AND variation (e.g. standard deviation) or associated estimates of uncertainty (e.g. confidence intervals) |
| ☐ | ☒ | For null hypothesis testing, the test statistic (e.g. *F*, *t*, *r*) with confidence intervals, effect sizes, degrees of freedom and *P* value noted<br>*Give P values as exact values whenever suitable.* |
| ☒ | ☐ | For Bayesian analysis, information on the choice of priors and Markov chain Monte Carlo settings |
| ☒ | ☐ | For hierarchical and complex designs, identification of the appropriate level for tests and full reporting of outcomes |
| ☐ | ☒ | Estimates of effect sizes (e.g. Cohen's *d*, Pearson's *r*), indicating how they were calculated |

*Our web collection on statistics for biologists contains articles on many of the points above.*

## Software and code

Policy information about availability of computer code

| | |
|---|---|
| Data collection | No software was used for data collection. |
| Data analysis | MuTect2, Strelka v2.0.13, Oncotator v1.9.1, MutSig2CV, ichorCNA v0.1.0, TitanCNA v1.17.1., GISTIC 2.0.22, STAR v2.5.3, RSEM v1.3.0, MiXCR 2.1.11, POLYSOLVER, LOHHLA. All custom code used in the analyses is available at https://github.com/jcarrotzhang/Code-for-preinvasive. |

For manuscripts utilizing custom algorithms or software that are central to the research but not yet described in published literature, software must be made available to editors/reviewers. We strongly encourage code deposition in a community repository (e.g. GitHub). See the Nature Research guidelines for submitting code & software for further information.

## Data

Policy information about availability of data

All manuscripts must include a data availability statement. This statement should provide the following information, where applicable:

- Accession codes, unique identifiers, or web links for publicly available datasets
- A list of figures that have associated raw data
- A description of any restrictions on data availability

Raw data from WES and RNA-seq of AIS/MIA and LUAD have been deposited in NODE (http://www.biosino.org/node) with accession code OEP000325.

# Field-specific reporting

Please select the one below that is the best fit for your research. If you are not sure, read the appropriate sections before making your selection.

☒ Life sciences ☐ Behavioural & social sciences ☐ Ecological, evolutionary & environmental sciences

For a reference copy of the document with all sections, see nature.com/documents/nr-reporting-summary-flat.pdf

# Life sciences study design

All studies must disclose on these points even when the disclosure is negative.

| | |
|---|---|
| Sample size | Sample size was not determined before the study. We studied 200 patients who underwent surgery between September 2011 and May 2016 at the Department of Thoracic Surgery, Fudan University Shanghai Cancer Center. Three samples were excluded due to failed sequencing quality control. |
| Data exclusions | Samples failed sequencing quality control were excluded. |
| Replication | Results are replicated using the TCGA data set as a validation cohort. |
| Randomization | Tumors were classified according to the lung adenocarcinoma classification of the International Association for the Study of Lung Cancer (IASLC), American Thoracic Society (ATS) and European Respiratory Society (ERS). |
| Blinding | Blinding was not relevant to our study. |

# Reporting for specific materials, systems and methods

We require information from authors about some types of materials, experimental systems and methods used in many studies. Here, indicate whether each material, system or method listed is relevant to your study. If you are not sure if a list item applies to your research, read the appropriate section before selecting a response.

## Materials & experimental systems

| n/a | Involved in the study | |
|---|---|---|
| ☒ | ☐ | Antibodies |
| ☒ | ☐ | Eukaryotic cell lines |
| ☒ | ☐ | Palaeontology |
| ☒ | ☐ | Animals and other organisms |
| ☐ | ☒ | Human research participants |
| ☒ | ☐ | Clinical data |

## Methods

| n/a | Involved in the study | |
|---|---|---|
| ☒ | ☐ | ChIP-seq |
| ☒ | ☐ | Flow cytometry |
| ☒ | ☐ | MRI-based neuroimaging |

## Human research participants

Policy information about studies involving human research participants

| | |
|---|---|
| Population characteristics | Detailed clinical characteristics of the patient cohort is shown in Supplementary Information Table 1. |
| Recruitment | Informed consents of all patients were obtained from patients themselves or their relatives. |
| Ethics oversight | This study was approved by the Committee for Ethical Review of Research (Fudan University Shanghai Cancer Center Institutional Review Board No. 090977-1). |

Note that full information on the approval of the study protocol must also be provided in the manuscript.
